# Supplementary material for: Modeling glioblastoma heterogeneity as a dynamic network of cell states
Source: Mol Syst Biol. 2021 Sep 16;17(9):e10105. doi: 10.15252/msb.202010105 (PMC8444284; doi:10.15252/msb.202010105)
Supplement: Supplementary file 5 — Source Data for Figure 3 [file MSB-17-e10105-s001.zip › Figure3A_sourcedata/GSEA_3065/nosigdb_state4.GseaPreranked.1622718430994/RICHARDS_NOWAKOWSKI_SCIENCE_2017_VRG_UPREG.html]

Details for gene set RICHARDS\_NOWAKOWSKI\_SCIENCE\_2017\_VRG\_UPREG[GSEA]

|  || Dataset | state4 |
| Phenotype | NoPhenotypeAvailable |
| Upregulated in class | na\_pos |
| GeneSet | RICHARDS\_NOWAKOWSKI\_SCIENCE\_2017\_VRG\_UPREG |
| Enrichment Score (ES) | 0.3538514 |
| Normalized Enrichment Score (NES) | 1.7366586 |
| Nominal p-value | 0.0 |
| FDR q-value | 0.014710019 |
| FWER p-Value | 0.137 |
Table: GSEA Results Summary

  

Fig 1: Enrichment plot: RICHARDS\_NOWAKOWSKI\_SCIENCE\_2017\_VRG\_UPREG      
 Profile of the Running ES Score & Positions of GeneSet Members on the Rank Ordered List

  

| PROBE | GENE SYMBOL | GENE\_TITLE | RANK IN GENE LIST | RANK METRIC SCORE | RUNNING ES | CORE ENRICHMENT || 1 | GFAP |  |  | 0 | 0.995 | 0.0295 | Yes |
| 2 | SAT1 |  |  | 4 | 0.669 | 0.0488 | Yes |
| 3 | FABP7 |  |  | 5 | 0.656 | 0.0683 | Yes |
| 4 | RAB31 |  |  | 21 | 0.388 | 0.0772 | Yes |
| 5 | FABP5 |  |  | 23 | 0.375 | 0.0882 | Yes |
| 6 | HOPX |  |  | 28 | 0.360 | 0.0982 | Yes |
| 7 | QKI |  |  | 31 | 0.345 | 0.1080 | Yes |
| 8 | SH3BGRL |  |  | 32 | 0.340 | 0.1181 | Yes |
| 9 | SERF2 |  |  | 44 | 0.307 | 0.1253 | Yes |
| 10 | SOX9 |  |  | 48 | 0.299 | 0.1337 | Yes |
| 11 | SEPT7 |  |  | 57 | 0.292 | 0.1410 | Yes |
| 12 | DKK3 |  |  | 66 | 0.281 | 0.1480 | Yes |
| 13 | CTNNA1 |  |  | 71 | 0.274 | 0.1554 | Yes |
| 14 | LGALS3 |  |  | 89 | 0.259 | 0.1602 | Yes |
| 15 | CLU |  |  | 95 | 0.252 | 0.1668 | Yes |
| 16 | TTYH1 |  |  | 100 | 0.249 | 0.1735 | Yes |
| 17 | CHMP2A |  |  | 104 | 0.246 | 0.1803 | Yes |
| 18 | PEA15 |  |  | 112 | 0.243 | 0.1863 | Yes |
| 19 | REXO2 |  |  | 127 | 0.236 | 0.1910 | Yes |
| 20 | ZFP36L2 |  |  | 135 | 0.228 | 0.1966 | Yes |
| 21 | RPS27L |  |  | 149 | 0.223 | 0.2010 | Yes |
| 22 | GATM |  |  | 156 | 0.219 | 0.2065 | Yes |
| 23 | ATP1B2 |  |  | 159 | 0.217 | 0.2126 | Yes |
| 24 | CNN3 |  |  | 160 | 0.217 | 0.2190 | Yes |
| 25 | PCGF5 |  |  | 161 | 0.216 | 0.2254 | Yes |
| 26 | HSPB1 |  |  | 180 | 0.211 | 0.2286 | Yes |
| 27 | EVI5 |  |  | 195 | 0.205 | 0.2324 | Yes |
| 28 | SDCBP |  |  | 212 | 0.201 | 0.2356 | Yes |
| 29 | ZEB1 |  |  | 214 | 0.198 | 0.2413 | Yes |
| 30 | HIGD1A |  |  | 216 | 0.198 | 0.2470 | Yes |
| 31 | GNG12 |  |  | 231 | 0.190 | 0.2503 | Yes |
| 32 | ANXA2 |  |  | 237 | 0.189 | 0.2550 | Yes |
| 33 | BBX |  |  | 242 | 0.187 | 0.2599 | Yes |
| 34 | SEPT11 |  |  | 251 | 0.185 | 0.2640 | Yes |
| 35 | ZBTB20 |  |  | 275 | 0.180 | 0.2655 | Yes |
| 36 | SALL1 |  |  | 286 | 0.177 | 0.2691 | Yes |
| 37 | NFE2L2 |  |  | 288 | 0.176 | 0.2741 | Yes |
| 38 | LITAF |  |  | 295 | 0.174 | 0.2782 | Yes |
| 39 | TRIM9 |  |  | 309 | 0.172 | 0.2812 | Yes |
| 40 | PDCD6 |  |  | 310 | 0.172 | 0.2863 | Yes |
| 41 | SEPT2 |  |  | 337 | 0.168 | 0.2869 | Yes |
| 42 | GPM6B |  |  | 346 | 0.166 | 0.2904 | Yes |
| 43 | RHOQ |  |  | 430 | 0.147 | 0.2808 | Yes |
| 44 | ABAT |  |  | 435 | 0.145 | 0.2844 | Yes |
| 45 | MYL6 |  |  | 436 | 0.145 | 0.2888 | Yes |
| 46 | KIF1B |  |  | 437 | 0.145 | 0.2931 | Yes |
| 47 | CTDSP2 |  |  | 444 | 0.144 | 0.2963 | Yes |
| 48 | SRI |  |  | 458 | 0.143 | 0.2984 | Yes |
| 49 | PHLDA1 |  |  | 464 | 0.142 | 0.3017 | Yes |
| 50 | SPAG16 |  |  | 491 | 0.139 | 0.3015 | Yes |
| 51 | ZFP36L1 |  |  | 497 | 0.137 | 0.3047 | Yes |
| 52 | PPP1CB |  |  | 504 | 0.136 | 0.3077 | Yes |
| 53 | JUND |  |  | 513 | 0.135 | 0.3103 | Yes |
| 54 | IFITM3 |  |  | 546 | 0.132 | 0.3089 | Yes |
| 55 | PPP2CB |  |  | 588 | 0.127 | 0.3057 | Yes |
| 56 | MLC1 |  |  | 594 | 0.126 | 0.3086 | Yes |
| 57 | PSAP |  |  | 596 | 0.126 | 0.3121 | Yes |
| 58 | HMGN3 |  |  | 598 | 0.125 | 0.3157 | Yes |
| 59 | NDUFB5 |  |  | 614 | 0.124 | 0.3168 | Yes |
| 60 | PTPN13 |  |  | 631 | 0.122 | 0.3178 | Yes |
| 61 | IRF2BP2 |  |  | 655 | 0.120 | 0.3174 | Yes |
| 62 | TAGLN2 |  |  | 672 | 0.118 | 0.3182 | Yes |
| 63 | MYO10 |  |  | 673 | 0.118 | 0.3217 | Yes |
| 64 | CASC10 |  |  | 682 | 0.117 | 0.3238 | Yes |
| 65 | FAM114A1 |  |  | 685 | 0.116 | 0.3269 | Yes |
| 66 | PHLPP1 |  |  | 740 | 0.109 | 0.3211 | Yes |
| 67 | ZFHX4 |  |  | 744 | 0.109 | 0.3238 | Yes |
| 68 | GSTP1 |  |  | 754 | 0.108 | 0.3255 | Yes |
| 69 | NADK2 |  |  | 766 | 0.106 | 0.3268 | Yes |
| 70 | SCRN1 |  |  | 767 | 0.106 | 0.3299 | Yes |
| 71 | LIPG |  |  | 771 | 0.106 | 0.3326 | Yes |
| 72 | CST3 |  |  | 792 | 0.104 | 0.3323 | Yes |
| 73 | PDCD4 |  |  | 796 | 0.104 | 0.3349 | Yes |
| 74 | C1orf61 |  |  | 803 | 0.104 | 0.3369 | Yes |
| 75 | ANXA5 |  |  | 814 | 0.103 | 0.3383 | Yes |
| 76 | FOS |  |  | 832 | 0.101 | 0.3384 | Yes |
| 77 | SHROOM3 |  |  | 855 | 0.099 | 0.3377 | Yes |
| 78 | TRPS1 |  |  | 858 | 0.099 | 0.3403 | Yes |
| 79 | COL11A1 |  |  | 905 | 0.095 | 0.3353 | Yes |
| 80 | PTGFRN |  |  | 941 | 0.092 | 0.3322 | Yes |
| 81 | SOX2 |  |  | 975 | 0.090 | 0.3293 | Yes |
| 82 | HEATR5A |  |  | 988 | 0.089 | 0.3299 | Yes |
| 83 | FAM19A5 |  |  | 1005 | 0.088 | 0.3298 | Yes |
| 84 | HTRA1 |  |  | 1008 | 0.088 | 0.3321 | Yes |
| 85 | MAPK1 |  |  | 1014 | 0.087 | 0.3338 | Yes |
| 86 | APC |  |  | 1029 | 0.086 | 0.3340 | Yes |
| 87 | SEMA5A |  |  | 1036 | 0.085 | 0.3355 | Yes |
| 88 | DOK5 |  |  | 1052 | 0.084 | 0.3355 | Yes |
| 89 | NOTCH3 |  |  | 1061 | 0.084 | 0.3366 | Yes |
| 90 | DTNA |  |  | 1063 | 0.084 | 0.3389 | Yes |
| 91 | SLC1A3 |  |  | 1069 | 0.083 | 0.3406 | Yes |
| 92 | SEMA6A |  |  | 1073 | 0.083 | 0.3425 | Yes |
| 93 | EMC2 |  |  | 1076 | 0.083 | 0.3446 | Yes |
| 94 | SRGAP3 |  |  | 1085 | 0.083 | 0.3458 | Yes |
| 95 | SEPT3 |  |  | 1113 | 0.081 | 0.3436 | Yes |
| 96 | AIF1L |  |  | 1119 | 0.081 | 0.3452 | Yes |
| 97 | SIPA1L1 |  |  | 1130 | 0.080 | 0.3459 | Yes |
| 98 | DDAH1 |  |  | 1153 | 0.078 | 0.3445 | Yes |
| 99 | CTBP2 |  |  | 1155 | 0.078 | 0.3466 | Yes |
| 100 | RFTN2 |  |  | 1157 | 0.078 | 0.3488 | Yes |
| 101 | SMAD5 |  |  | 1186 | 0.077 | 0.3463 | Yes |
| 102 | ETV1 |  |  | 1199 | 0.076 | 0.3466 | Yes |
| 103 | CA12 |  |  | 1205 | 0.076 | 0.3480 | Yes |
| 104 | TTYH3 |  |  | 1206 | 0.076 | 0.3502 | Yes |
| 105 | GAB1 |  |  | 1209 | 0.076 | 0.3521 | Yes |
| 106 | TFPI |  |  | 1213 | 0.075 | 0.3539 | Yes |
| 107 | KCNN3 |  |  | 1238 | 0.074 | 0.3520 | No |
| 108 | DOCK1 |  |  | 1252 | 0.073 | 0.3520 | No |
| 109 | ARHGAP5 |  |  | 1259 | 0.073 | 0.3531 | No |
| 110 | BICD1 |  |  | 1271 | 0.072 | 0.3534 | No |
| 111 | GLUD1 |  |  | 1295 | 0.071 | 0.3516 | No |
| 112 | CHP1 |  |  | 1347 | 0.069 | 0.3451 | No |
| 113 | VIM |  |  | 1350 | 0.069 | 0.3468 | No |
| 114 | FAM171B |  |  | 1353 | 0.068 | 0.3485 | No |
| 115 | APBB2 |  |  | 1358 | 0.068 | 0.3498 | No |
| 116 | FSTL1 |  |  | 1367 | 0.068 | 0.3505 | No |
| 117 | C1orf198 |  |  | 1394 | 0.067 | 0.3481 | No |
| 118 | SPAG9 |  |  | 1420 | 0.065 | 0.3458 | No |
| 119 | FERMT2 |  |  | 1426 | 0.065 | 0.3469 | No |
| 120 | FZD3 |  |  | 1429 | 0.065 | 0.3485 | No |
| 121 | HES1 |  |  | 1442 | 0.065 | 0.3484 | No |
| 122 | NPAS3 |  |  | 1483 | 0.062 | 0.3435 | No |
| 123 | TRIB2 |  |  | 1496 | 0.062 | 0.3433 | No |
| 124 | LRP4 |  |  | 1502 | 0.062 | 0.3443 | No |
| 125 | LHX2 |  |  | 1534 | 0.061 | 0.3409 | No |
| 126 | MT2A |  |  | 1542 | 0.060 | 0.3415 | No |
| 127 | NES |  |  | 1551 | 0.060 | 0.3419 | No |
| 128 | SEPT9 |  |  | 1564 | 0.059 | 0.3416 | No |
| 129 | TSPAN3 |  |  | 1584 | 0.059 | 0.3402 | No |
| 130 | ZC3HAV1 |  |  | 1592 | 0.059 | 0.3408 | No |
| 131 | REST |  |  | 1593 | 0.059 | 0.3425 | No |
| 132 | GLI3 |  |  | 1603 | 0.059 | 0.3427 | No |
| 133 | MRC2 |  |  | 1647 | 0.057 | 0.3372 | No |
| 134 | PELI2 |  |  | 1651 | 0.057 | 0.3383 | No |
| 135 | TOB2 |  |  | 1656 | 0.057 | 0.3394 | No |
| 136 | IQSEC1 |  |  | 1670 | 0.056 | 0.3388 | No |
| 137 | TGIF1 |  |  | 1699 | 0.055 | 0.3357 | No |
| 138 | GNG5 |  |  | 1709 | 0.055 | 0.3359 | No |
| 139 | DMD |  |  | 1710 | 0.055 | 0.3375 | No |
| 140 | ZMAT1 |  |  | 1718 | 0.055 | 0.3379 | No |
| 141 | MSI1 |  |  | 1744 | 0.054 | 0.3353 | No |
| 142 | ITGB8 |  |  | 1765 | 0.053 | 0.3335 | No |
| 143 | EEF1D |  |  | 1766 | 0.053 | 0.3351 | No |
| 144 | CKB |  |  | 1774 | 0.053 | 0.3355 | No |
| 145 | ALDH6A1 |  |  | 1803 | 0.052 | 0.3323 | No |
| 146 | STXBP3 |  |  | 1804 | 0.052 | 0.3339 | No |
| 147 | CTNND1 |  |  | 1840 | 0.051 | 0.3295 | No |
| 148 | ARAP2 |  |  | 1845 | 0.051 | 0.3303 | No |
| 149 | RGL1 |  |  | 1927 | 0.049 | 0.3181 | No |
| 150 | PALLD |  |  | 1930 | 0.049 | 0.3192 | No |
| 151 | DDR1 |  |  | 1941 | 0.048 | 0.3190 | No |
| 152 | LPIN2 |  |  | 1944 | 0.048 | 0.3201 | No |
| 153 | LIFR |  |  | 1972 | 0.048 | 0.3170 | No |
| 154 | SLC35F1 |  |  | 1996 | 0.047 | 0.3145 | No |
| 155 | GRB14 |  |  | 2026 | 0.046 | 0.3110 | No |
| 156 | NRBP2 |  |  | 2041 | 0.046 | 0.3100 | No |
| 157 | SYNE1 |  |  | 2057 | 0.045 | 0.3088 | No |
| 158 | PDLIM3 |  |  | 2070 | 0.045 | 0.3081 | No |
| 159 | HEG1 |  |  | 2075 | 0.045 | 0.3087 | No |
| 160 | OAT |  |  | 2106 | 0.044 | 0.3050 | No |
| 161 | PREX1 |  |  | 2110 | 0.044 | 0.3058 | No |
| 162 | SHC3 |  |  | 2114 | 0.043 | 0.3065 | No |
| 163 | PSAT1 |  |  | 2153 | 0.043 | 0.3014 | No |
| 164 | SEL1L3 |  |  | 2171 | 0.042 | 0.2998 | No |
| 165 | LARP7 |  |  | 2188 | 0.042 | 0.2983 | No |
| 166 | FAM219A |  |  | 2194 | 0.042 | 0.2987 | No |
| 167 | ACBD5 |  |  | 2221 | 0.041 | 0.2956 | No |
| 168 | SPRED1 |  |  | 2272 | 0.040 | 0.2883 | No |
| 169 | PHYHIPL |  |  | 2328 | 0.038 | 0.2802 | No |
| 170 | CCND2 |  |  | 2367 | 0.038 | 0.2749 | No |
| 171 | SPRY2 |  |  | 2432 | 0.036 | 0.2652 | No |
| 172 | TSPAN6 |  |  | 2443 | 0.036 | 0.2646 | No |
| 173 | RFX4 |  |  | 2454 | 0.036 | 0.2640 | No |
| 174 | PDLIM5 |  |  | 2517 | 0.034 | 0.2546 | No |
| 175 | MID1 |  |  | 2518 | 0.034 | 0.2556 | No |
| 176 | HSDL2 |  |  | 2546 | 0.034 | 0.2520 | No |
| 177 | NCKAP5 |  |  | 2563 | 0.034 | 0.2503 | No |
| 178 | LRIG3 |  |  | 2573 | 0.033 | 0.2498 | No |
| 179 | TMEM170A |  |  | 2589 | 0.033 | 0.2483 | No |
| 180 | RHOJ |  |  | 2605 | 0.033 | 0.2467 | No |
| 181 | SPATA13 |  |  | 2617 | 0.033 | 0.2458 | No |
| 182 | HMGCS1 |  |  | 2674 | 0.031 | 0.2373 | No |
| 183 | CALD1 |  |  | 2682 | 0.031 | 0.2370 | No |
| 184 | VCL |  |  | 2710 | 0.030 | 0.2334 | No |
| 185 | BMPR1B |  |  | 2711 | 0.030 | 0.2343 | No |
| 186 | IDI1 |  |  | 2749 | 0.030 | 0.2289 | No |
| 187 | ABCD3 |  |  | 2800 | 0.029 | 0.2214 | No |
| 188 | SGSM2 |  |  | 2873 | 0.027 | 0.2100 | No |
| 189 | SRGAP2C |  |  | 2902 | 0.027 | 0.2061 | No |
| 190 | STK39 |  |  | 2943 | 0.026 | 0.2001 | No |
| 191 | MGST3 |  |  | 2993 | 0.025 | 0.1926 | No |
| 192 | CELSR1 |  |  | 2995 | 0.025 | 0.1932 | No |
| 193 | AEBP2 |  |  | 3031 | 0.024 | 0.1880 | No |
| 194 | YBX3 |  |  | 3049 | 0.024 | 0.1859 | No |
| 195 | ZCCHC3 |  |  | 3063 | 0.024 | 0.1844 | No |
| 196 | STX16 |  |  | 3067 | 0.024 | 0.1846 | No |
| 197 | ATXN2 |  |  | 3075 | 0.024 | 0.1841 | No |
| 198 | GCA |  |  | 3079 | 0.023 | 0.1843 | No |
| 199 | LRIG1 |  |  | 3101 | 0.023 | 0.1814 | No |
| 200 | PHACTR2 |  |  | 3125 | 0.023 | 0.1782 | No |
| 201 | PTAR1 |  |  | 3126 | 0.023 | 0.1789 | No |
| 202 | F3 |  |  | 3133 | 0.022 | 0.1786 | No |
| 203 | IL6ST |  |  | 3218 | 0.021 | 0.1650 | No |
| 204 | MSI2 |  |  | 3242 | 0.020 | 0.1617 | No |
| 205 | MEGF10 |  |  | 3315 | 0.018 | 0.1501 | No |
| 206 | CTNNB1 |  |  | 3329 | 0.017 | 0.1485 | No |
| 207 | CDO1 |  |  | 3358 | 0.017 | 0.1442 | No |
| 208 | GPC4 |  |  | 3380 | 0.016 | 0.1412 | No |
| 209 | LSS |  |  | 3382 | 0.016 | 0.1415 | No |
| 210 | IGDCC4 |  |  | 3402 | 0.016 | 0.1387 | No |
| 211 | MYO6 |  |  | 3404 | 0.015 | 0.1390 | No |
| 212 | AKAP13 |  |  | 3405 | 0.015 | 0.1395 | No |
| 213 | RDX |  |  | 3422 | 0.015 | 0.1372 | No |
| 214 | TPI1 |  |  | 3442 | 0.014 | 0.1345 | No |
| 215 | PLCE1 |  |  | 3446 | 0.014 | 0.1344 | No |
| 216 | TJP1 |  |  | 3531 | 0.011 | 0.1205 | No |
| 217 | ITM2C |  |  | 3587 | 0.009 | 0.1116 | No |
| 218 | TMEM132B |  |  | 3600 | 0.009 | 0.1098 | No |
| 219 | PCMTD1 |  |  | 3716 | 0.004 | 0.0906 | No |
| 220 | H6PD |  |  | 3736 | 0.004 | 0.0875 | No |
| 221 | MSN |  |  | 3767 | 0.002 | 0.0825 | No |
| 222 | AGO3 |  |  | 3784 | 0.001 | 0.0798 | No |
| 223 | RAB3GAP2 |  |  | 3789 | 0.001 | 0.0792 | No |
| 224 | CD99 |  |  | 3814 | 0.000 | 0.0751 | No |
| 225 | DARS |  |  | 3886 | -0.003 | 0.0633 | No |
| 226 | CUL3 |  |  | 3899 | -0.003 | 0.0614 | No |
| 227 | KLF6 |  |  | 3920 | -0.005 | 0.0581 | No |
| 228 | STAT3 |  |  | 3937 | -0.006 | 0.0556 | No |
| 229 | NOTCH2 |  |  | 3944 | -0.006 | 0.0548 | No |
| 230 | TLK1 |  |  | 3945 | -0.006 | 0.0550 | No |
| 231 | NTRK3 |  |  | 3955 | -0.007 | 0.0537 | No |
| 232 | JUN |  |  | 3979 | -0.008 | 0.0500 | No |
| 233 | EGR1 |  |  | 3998 | -0.010 | 0.0473 | No |
| 234 | FDFT1 |  |  | 4088 | -0.014 | 0.0327 | No |
| 235 | SAMD4A |  |  | 4118 | -0.017 | 0.0283 | No |
| 236 | DBI |  |  | 4122 | -0.017 | 0.0283 | No |
| 237 | VCAN |  |  | 4158 | -0.019 | 0.0230 | No |
| 238 | MEIS1 |  |  | 4218 | -0.023 | 0.0138 | No |
| 239 | PDPN |  |  | 4220 | -0.023 | 0.0143 | No |
| 240 | EIF2AK2 |  |  | 4256 | -0.025 | 0.0091 | No |
| 241 | PAG1 |  |  | 4261 | -0.026 | 0.0092 | No |
| 242 | LPP |  |  | 4265 | -0.026 | 0.0095 | No |
| 243 | FKBP9 |  |  | 4273 | -0.027 | 0.0091 | No |
| 244 | ATP2B4 |  |  | 4292 | -0.028 | 0.0069 | No |
| 245 | DAG1 |  |  | 4317 | -0.030 | 0.0038 | No |
| 246 | NOTCH1 |  |  | 4321 | -0.031 | 0.0042 | No |
| 247 | ID4 |  |  | 4329 | -0.031 | 0.0039 | No |
| 248 | EZR |  |  | 4342 | -0.032 | 0.0029 | No |
| 249 | ARL6IP5 |  |  | 4364 | -0.033 | 0.0003 | No |
| 250 | REEP3 |  |  | 4373 | -0.034 | -0.0001 | No |
| 251 | SCAF11 |  |  | 4415 | -0.037 | -0.0059 | No |
| 252 | FLCN |  |  | 4427 | -0.037 | -0.0066 | No |
| 253 | TMEM98 |  |  | 4455 | -0.041 | -0.0100 | No |
| 254 | EFNB2 |  |  | 4534 | -0.047 | -0.0217 | No |
| 255 | STAG2 |  |  | 4558 | -0.049 | -0.0241 | No |
| 256 | FGFR1 |  |  | 4568 | -0.049 | -0.0242 | No |
| 257 | SYT11 |  |  | 4599 | -0.051 | -0.0277 | No |
| 258 | FKBP10 |  |  | 4638 | -0.054 | -0.0325 | No |
| 259 | GLO1 |  |  | 4640 | -0.054 | -0.0311 | No |
| 260 | RYK |  |  | 4685 | -0.058 | -0.0368 | No |
| 261 | NAPEPLD |  |  | 4707 | -0.060 | -0.0385 | No |
| 262 | SCD |  |  | 4717 | -0.061 | -0.0382 | No |
| 263 | BMP7 |  |  | 4727 | -0.062 | -0.0379 | No |
| 264 | IFI6 |  |  | 4742 | -0.063 | -0.0384 | No |
| 265 | STK17A |  |  | 4774 | -0.066 | -0.0417 | No |
| 266 | TFAP2C |  |  | 4780 | -0.066 | -0.0405 | No |
| 267 | ENO1 |  |  | 4868 | -0.073 | -0.0530 | No |
| 268 | PTPRG |  |  | 4952 | -0.079 | -0.0647 | No |
| 269 | SNX13 |  |  | 4971 | -0.080 | -0.0653 | No |
| 270 | MEIS2 |  |  | 5012 | -0.084 | -0.0696 | No |
| 271 | CSMD2 |  |  | 5016 | -0.084 | -0.0676 | No |
| 272 | PPA1 |  |  | 5017 | -0.084 | -0.0651 | No |
| 273 | UBXN4 |  |  | 5034 | -0.086 | -0.0653 | No |
| 274 | JADE1 |  |  | 5070 | -0.089 | -0.0685 | No |
| 275 | TMED10 |  |  | 5078 | -0.089 | -0.0671 | No |
| 276 | CARHSP1 |  |  | 5107 | -0.092 | -0.0691 | No |
| 277 | ROBO1 |  |  | 5126 | -0.094 | -0.0693 | No |
| 278 | AASS |  |  | 5154 | -0.097 | -0.0710 | No |
| 279 | LRP6 |  |  | 5164 | -0.098 | -0.0696 | No |
| 280 | JAM2 |  |  | 5167 | -0.099 | -0.0670 | No |
| 281 | PHIP |  |  | 5215 | -0.103 | -0.0719 | No |
| 282 | ELOVL5 |  |  | 5226 | -0.103 | -0.0705 | No |
| 283 | FGFBP3 |  |  | 5233 | -0.104 | -0.0684 | No |
| 284 | CLDND1 |  |  | 5238 | -0.104 | -0.0660 | No |
| 285 | PON2 |  |  | 5246 | -0.105 | -0.0640 | No |
| 286 | PTN |  |  | 5261 | -0.107 | -0.0632 | No |
| 287 | DAD1 |  |  | 5287 | -0.109 | -0.0642 | No |
| 288 | ITGAV |  |  | 5309 | -0.110 | -0.0645 | No |
| 289 | PUS7 |  |  | 5310 | -0.110 | -0.0612 | No |
| 290 | TNC |  |  | 5359 | -0.114 | -0.0659 | No |
| 291 | JAM3 |  |  | 5414 | -0.120 | -0.0715 | No |
| 292 | LPCAT1 |  |  | 5437 | -0.122 | -0.0716 | No |
| 293 | IQGAP2 |  |  | 5451 | -0.125 | -0.0701 | No |
| 294 | CAPN2 |  |  | 5478 | -0.129 | -0.0706 | No |
| 295 | RBM39 |  |  | 5499 | -0.131 | -0.0701 | No |
| 296 | ITGA6 |  |  | 5574 | -0.139 | -0.0784 | No |
| 297 | CNTNAP3B |  |  | 5581 | -0.140 | -0.0753 | No |
| 298 | CREB5 |  |  | 5611 | -0.146 | -0.0759 | No |
| 299 | SLC25A37 |  |  | 5639 | -0.151 | -0.0759 | No |
| 300 | DNAJC1 |  |  | 5696 | -0.160 | -0.0806 | No |
| 301 | CACYBP |  |  | 5713 | -0.162 | -0.0785 | No |
| 302 | TSPAN5 |  |  | 5716 | -0.163 | -0.0740 | No |
| 303 | CDCA7L |  |  | 5720 | -0.164 | -0.0697 | No |
| 304 | SGCE |  |  | 5743 | -0.171 | -0.0683 | No |
| 305 | CANX |  |  | 5755 | -0.173 | -0.0650 | No |
| 306 | TMX1 |  |  | 5759 | -0.174 | -0.0604 | No |
| 307 | ACTN1 |  |  | 5765 | -0.176 | -0.0560 | No |
| 308 | BARD1 |  |  | 5779 | -0.179 | -0.0529 | No |
| 309 | SLC12A2 |  |  | 5790 | -0.181 | -0.0492 | No |
| 310 | SLC16A1 |  |  | 5794 | -0.182 | -0.0443 | No |
| 311 | PTPRZ1 |  |  | 5811 | -0.186 | -0.0415 | No |
| 312 | AXL |  |  | 5826 | -0.189 | -0.0383 | No |
| 313 | LDLR |  |  | 5843 | -0.194 | -0.0352 | No |
| 314 | TMBIM6 |  |  | 5855 | -0.196 | -0.0313 | No |
| 315 | MSMO1 |  |  | 5866 | -0.198 | -0.0271 | No |
| 316 | FAT1 |  |  | 5898 | -0.206 | -0.0262 | No |
| 317 | HMGCR |  |  | 5906 | -0.208 | -0.0212 | No |
| 318 | WEE1 |  |  | 5907 | -0.209 | -0.0150 | No |
| 319 | DHCR24 |  |  | 5943 | -0.219 | -0.0145 | No |
| 320 | RCN1 |  |  | 5945 | -0.219 | -0.0081 | No |
| 321 | MAGI1 |  |  | 5985 | -0.233 | -0.0078 | No |
| 322 | AKAP12 |  |  | 5988 | -0.234 | -0.0012 | No |
| 323 | MLEC |  |  | 5997 | -0.237 | 0.0044 | No |
| 324 | EMP2 |  |  | 6032 | -0.254 | 0.0063 | No |
| 325 | SYNE2 |  |  | 6086 | -0.287 | 0.0058 | No |
| 326 | SQLE |  |  | 6123 | -0.316 | 0.0092 | No |
| 327 | BCAN |  |  | 6211 | -0.481 | 0.0088 | No |
Table: GSEA details [plain text format]

  

Fig 2: RICHARDS\_NOWAKOWSKI\_SCIENCE\_2017\_VRG\_UPREG: Random ES distribution      
 Gene set null distribution of ES for **RICHARDS\_NOWAKOWSKI\_SCIENCE\_2017\_VRG\_UPREG**
